# What is holding back business process virtualization in the post-COVID-19 era? Based on process virtualization theory (PVT)

**DOI:** 10.3389/fpsyg.2023.1084180

**Published:** 2023-02-15

**Authors:** Yituo Feng, Jungryeol Park, Miao Feng

**Affiliations:** ^1^Management Information Systems, Chungbuk National University, Cheongju, Republic of Korea; ^2^Technology Policy Research Division, Electronics and Telecommunications Research Institute (ETRI), Daejeon, Republic of Korea; ^3^Business School, Shandong Management University, Jinan, China

**Keywords:** telecommuting, collaboration software, process virtualization theory (PVT), business process virtualizability, post COVID-19 era

## Abstract

The post-pandemic COVID-19 has been influential in accelerating the digital transformation of enterprises and business process virtualization. However, in a virtual working environment with no physical interaction, the psychological requirements of the communication between teleworkers and the negative impact of information systems are hindering the business process virtualization. Studying the relationship between the interaction between organizational members and job performance is an important part of organizational psychology. For an enterprise to maintain high-efficiency output, it is necessary to study psychological factors related to business process virtualization. This paper verified the factors hindering business process virtualization based on process virtualization theory (PVT). The research was implemented on a sample of 343 teleworkers in China enterprises. The structure of the model of this study includes two aspects that hinder the business process virtualization: the psychological requirements of teleworkers (Sensory requirements, Synchronism requirements, and Relationship requirements) and the negative effects of information systems (Information overload and Communication overload). The results show that teleworkers’ sensory requirements, synchronism requirements, and communication overload negatively impact business process virtualization. However, unlike the results in the existing literature, the relationship requirements and information overload do not affect the business process virtualization. The results will help business managers, teleworkers, and information system developers develop strategies to address the negative factors hindering business process virtualization. In the so-called new “normal era,” our research will help companies to create a successful virtual work environment.

## Introduction

In the modern world, some processes that used to take place face-to-face in physical environments are now in contactless virtual spaces. Due to the rapid development of ICT technology and information systems, some processes can work equally well in a virtual environment without face-to-face interaction. For example, face-to-face education is shifting to distance learning as correspondence education (Alarabiat et al., 2023), offline shopping is shifting to e-commerce (Overby, 2012), the process of consumers interacting with brands is changing to a virtual brand community (Jing et al., 2017), and offline social activities between people are shifting to social activities in the virtual world (Cross et al., 2016). In the field of artificial intelligence (AI), Scholars studying AI have built a Visual Question Answering (VQA) model based on natural language processing tasks, which optimizes the virtualization of language translation and text question-answering processes (Zheng et al., 2021c). In addition, computers learn to perform semantic expression, image recognition, and visual reasoning like humans through deep learning. In the future, human reasoning and thinking processes may become increasingly virtualized through AI technology (Zheng et al., 2021a,b).

In the commercial field, business processes in physical space are migrating to a virtual space based on information systems. This migration process is called business process virtualization. The business process virtualization of organizations is to alleviate energy problems and achieve carbon neutrality and business continuity (Caldow, 2009; Heng et al., 2012; Huang and Liu, 2021). For this reason, companies introduce collaboration software to break through work’s time and space constraints (Goluboff, 2001; Shockley and Allen, 2007; Tworoger et al., 2013). Especially since countries around the world have recently started working remotely due to COVID-19, this business process virtualization is accelerating. In China, the number of people working remotely through collaboration software such as DingTalk increased from 4.9 million in 2018 to 2020, More than 300 million, an increase of about five times (Cheng and Zhang, 2022). DingTalk is work-oriented social media to improve team and employee performance (Song et al., 2019). The explosive growth of collaboration software is that business managers want to use collaboration software to move business processes into a virtual space and positively impact the business.

However, some research related to collaboration software shows that business process virtualization is not progressing smoothly, and the introduction of collaboration software has yet to achieve higher work performance for companies (Golden and Veiga, 2005, 2008; Wang et al., 2008; Cross et al., 2016; Giermindl et al., 2018a,b; Ammirato et al., 2019). So not all processes can be well virtualized (Overby, 2008; Overby and Konsynski, 2010; Graupner and Maedche, 2015; Alarabiat et al., 2023). Business processes in a physical environment face hurdles in migrating to a virtualized environment based on information systems. These hurdles are not conducive for enterprises to business process virtualization. The existing literature has been extensively discussed through various theoretical perspectives to weigh the benefits and challenges of information system-based telecommuting, such as the Technology Acceptance Model (Silva-C, 2019; Donati et al., 2021; Chai et al., 2022), Innovation Diffusion Theory (Al-Rahmi et al., 2019, 2021; Tsai and Hung, 2020), and Task-Technology Fit Theory (Al-Rahmi et al., 2022; Huang and Wang, 2022). These theoretical lenses make significant contributions at the level of individual acceptance of information systems. However, there are some gaps in measuring the level of business process virtualization and the psychological requirements of teleworkers. As employees become more digitally literate, organizational members are becoming more sophisticated about using collaboration software (Reddy et al., 2020). Factors at the level of psychological requirements caused by changes in the workplace may have a more profound impact on business process virtualization than factors at the technical level. For example, the relationship formed offline face-to-face is stronger than the relationship formed online (Mesch and Talmud, 2006). Therefore, business processes may not be suitable for virtualization for employees with high relationship requirements. However, few telecommuting studies have explored these factors from a process virtualization theory (PVT) perspective. In the future, in the face of an increasingly virtualized society, we need research to help enterprises improve the virtualization of business processes.

This study aims to discover the critical factors that hinder business process virtualization based on PVT. According to the research results, it provides enterprises with feasible management suggestions and helps them migrate their business processes to the virtual environment. The PVT explains how a process in a physical environment migrates to a virtual environment based on information systems (Overby, 2008). PVT demonstrates that psychological needs significantly and negatively affect process virtualization but that positive effects of information systems moderate these negative effects. However, a key problem with much of the literature regarding PVT is that it needs to pay attention to the negative effects of information systems (Alarabiat et al., 2023). After business processes are migrated to an information system-based virtual space, the quantity and frequency of information exchange will increase, further amplifying the adverse effects of information overload and communication overload (Granero-Gallegos et al., 2021; Barrett et al., 2022). It is necessary to explore the negative factors that information systems bring to business processes to improve the degree of business process virtualization. Therefore, this study proposes a quasi-business process virtualization model based on PVT to fill the research gap that ignores the adverse effects of information systems in PVT. We use a new model to examine the key factors hindering business process virtualization comprehensively.

The results of this study show that teleworkers’ sensory requirements, synchronism requirements, and communication overload are essential factors that negatively impact business process virtualization. The findings match earlier studies’ findings (Overby and Konsynski, 2010; Balci and Rosenkranz, 2014; Graupner and Maedche, 2015; Agrawal et al., 2020; Granero-Gallegos et al., 2021; Barrett et al., 2022; Alarabiat et al., 2023). However, unlike the results in the existing literature, in this study, the relationship requirements and information overload do not affect the business process virtualization. Factors that hinder business process virtualization change as information systems mature and organizations become more digitally literate. This study makes several theoretical and practical contributions. First, this study helps to apply PVT to research business processes and expands the scope of application of the theory. Second, it also discusses and verifies the negative impact of information systems and provides a new research perspective for scholars who study organizational psychology and develop information systems in the future. Ultimately, the results will help businesses develop strategies to create successful virtual work environments.

## Theoretical background

### Process virtualization theory

Overby (2008, 2012) defines this transition from a physical process to a virtual process as process virtualization. As an emerging phenomenon in information systems, Overby (2008, 2012) proposed PVT to explain the process virtualization. The focus of PVT is to understand how and to what extent a process that operates in a face-to-face physical environment can be implemented in a contactless virtual environment (Alarabiat et al., 2023). Overby (2008) defined the expected effect or actual result obtained by promoting process virtualization as process virtualizability and presented a dependent variable (process virtualizability) to explain and predict how suitable a process is to be performed in a virtual environment.

Overby (2008) first proposed that the PVT includes two groups of independent variables. The first is the psychological requirements group of the business process, and the second is the IT characteristic group, which affects process virtualizability. The psychological requirements group has sensory, relationship, synchronism, and identification and control requirements. Sensory requirements refer to the fact that participants in a virtual process need to be able to enjoy the whole sensory experience in the traditional physical environment. Synchronism requirements refer to the requirements that participants in a virtual process need to communicate with other participants with the lowest delay. Relationship requirements refer to the perceived needs of participants in a virtual process to interact, socialize, and establish friendships with other process participants. Identification and control requirements refer to the requirements of participants in a virtual process to identify other participants in the same process. These psychological requirements negatively impact process virtualizability. The IT characteristic group has monitoring capability, reach, and richness. Monitoring Capability refers to the ability of an information system to provide high-quality information. Reach refers to the ability provided by information systems to break through time and distance constraints. Richness refers to the ability of an information system to provide rich and diverse information. The IT characteristics can mitigate the negative impact of process requirements on process virtualizability.

In subsequent studies, the definition of process virtualizability is slightly different depending on the researcher. Overby and Konsynski (2010) defined process virtualizability as the extent to which they can be easily performed between people or between people and objects without physical interaction. Balci and Rosenkranz (2014) defines process virtualizability as the degree to which it is possible to succeed in converting a process from a physical environment to a virtual environment. Alarabiat et al. (2023) defines process virtualizability as the adaptability of transferring from the physical environment to the virtual environment at any time.

Furthermore, more researchers demonstrated the advancement of PVT application in different scholarly contexts (Overby and Konsynski, 2010; Barth and Veit, 2011; Balci and Rosenkranz, 2014; Mburu and Oboko, 2018; Agrawal et al., 2020; Alarabiat et al., 2023). Czarnecki et al. (2010) propose a framework for planning the processes virtualization intended to assist the decision-maker in prioritizing the processes to be virtualized: highlighting their demand for changes at different levels of the organization. Based on the theoretical foundation of PVT, Graupner and Maedche (2015) conducted a quantitative study to identify the factors influencing intended digital process use in retail banking. The results indicate that relationship, sensory, and control requirements inhibit intended digital process use in retail banking. The results not only contribute to a better understanding of the factors that influence intended digital process use in retail banking but also provide an empirical examination of a theory that has been largely untested. Jasper (2015, 2019) integrates developed new product features into a joint virtual reality (VR)-based environment for measuring consumer preferences during product innovation development. Virtualization of the new product demonstration process offers the potential for early customer integration in the new product development process. To assess whether IT-enabled process virtualization capabilities impact organizational Green IT initiatives, Tomás et al. (2018) proposed a conceptual model that combines three theories: technology-organization-environment framework, PVT, and diffusion of innovation theory. PVT can explain whether processes are suitable for migration into virtual environments, such as those enabled by information technology.

## Hypothesis development

### Psychological requirements and business process virtualizability

In this study, we hypothesize that psychological requirements (Sensory requirements, Synchronism requirements, and Relationship requirements) negatively impact business process virtualizability based on the basic PVT theoretical model. The following is the basis for our derivation of this hypothesis.

Sensory requirements are that organization members must be able to enjoy the whole sensory experience of traditional work processes (e.g., seeing and hearing other colleagues or leaders in the workplace and having a feeling of being in the office). In the virtual environment, the communication and interaction between people rely on IT technology and information systems, so it is impossible to fully experience the visual and olfactory sensory experience in the virtual environment. Therefore, if the sensory demands of the work to be performed are high, process virtualizability may be low. According to The Law of Mehrabian, if the importance of linguistic elements in communication is 7%, non-verbal elements such as gestures, tone, and intonation is 93% (Mehrabian, 1971). While group members can converse in a near-face-to-face feel via video conferencing, there are limits to capturing each other’s facial expressions, speech, and attitude changes in detail. Therefore, if members of the organization have high sensory demands on the work handled in the virtual environment, it may lead to poor communication and poor work processing (Overby and Konsynski, 2010; Balci and Rosenkranz, 2014). According to related research, higher sensory demands lead to lower process virtuality (Overby, 2008, 2012; Overby and Konsynski, 2010; Balci and Rosenkranz, 2014; Graupner and Maedche, 2015; Agrawal et al., 2020; Alarabiat et al., 2023). The present study builds on the hypothesis that sensory demands impair process virtuality based on previous studies on sensory demands.

H1: Sensory requirements negatively impact business process virtualizability.

Relationship requirements refer to the degree to which process participants need to interact socially with others involved in the process to meet professional needs (Overby, 2008; Overby and Konsynski, 2010; Balci and Rosenkranz, 2014; Graupner and Maedche, 2015; Alarabiat et al., 2023). This social interaction can foster friendships and knowledge sharing among organizational members, enhancing team creativity (Wang et al., 2021). The formation and development of relationships among organizational members or supervisors in the workplace is the focus of many scholars’ research. Fay and Kline (2012) believe that organizational members with high-quality friendships have higher organizational commitment and identification. In addition, high-quality supervisor relationships create cognitive and social resources that can satisfy the members’ sense of belonging (Bono and Yoon, 2012). However, telecommuting has changed how people communicate, damaging the quality of relationships between colleagues and supervisors. The reason is that this relationship is weaker in virtual environments than in face-to-face physical environments (Mesch and Talmud, 2006; Lima et al., 2017). According to related studies, higher relational needs lead to lower process virtualizability (Overby, 2008, 2012; Overby and Konsynski, 2010; Barth and Veit, 2011; Balci and Rosenkranz, 2014; Graupner and Maedche, 2015; Mburu and Oboko, 2018; Alarabiat et al., 2023). For example, Barth and Veit (2011) confirmed in a study on e-government services that the more demanding users need to establish relationships with responsible public officials, the lower the acceptance of online services. Graupner and Maedche (2015) found that bank customers’ intention to use online banking is lower if they have higher intentions to build relationships and develop friendships with bank staff. Alarabiat et al. (2023) confirmed in their research on online learning that if students need to interact and build relationships with their classmates or educators in a physical space, students are less satisfied with online learning platforms and decrease their intent to continue using online learning. Based on these results, an information system-based virtual environment may not be suitable if people involved in performing work need to build friendships and trust and accumulate the knowledge necessary for performing work. So, we propose the following hypothesis.

H2: Relationship requirements negatively impact business process virtualizability.

Synchronism requirements are the level at which members of an organization can communicate immediately with other people involved in a business process that needs to be processed immediately with minimal delay (Overby, 2008; Overby and Konsynski, 2010; Graupner and Maedche, 2015; Alarabiat et al., 2023). Teamwork is playing an increasingly important role in many jobs, and teamwork quality has always been a critical factor in project success and organizational performance (Gander et al., 2020). A high-quality team needs synchronous and efficient communication to achieve consensus within the team. According to the Media Synchronicity Theory proposed by Dennis et al. (2008), when two or more people are in the same business process, there is synchronization between them. Media Synchronicity Theory has verified five factors that affect the level of synchronization, including Transmission speed, Parallel processing, Symbol diversity, Rehearsal, and Re-processing. Communication among teleworkers using corporate social media can be broken down into two processes: conveyance and convergence. The conveyance process means a large amount of information is exchanged between workers, and the convergence process involves consensus on these messages. Higher synchrony among team members means smoother social interaction, which is conducive to improving the cohesive social capital of employees, all of which positively impact work efficiency (Jong et al., 2021). In the face-to-face physical space, organization members can see colleagues and communicate anytime, quickly exchanging the necessary information to meet people’s synchronization needs.

In contrast, in the environment of process virtualization, the organization’s members need to meet the synchronization requirements through means based on IT communication systems such as enterprise SNS. However, compared to a face-to-face physical environment, the synchronization process facilitated by IT may be subject to limitations due to factors that impact the level of synchronization, such as transmission speed, parallel processing capabilities, diversity of symbols, and opportunities for rehearsal. The presence of these limitations may impede the synchronization process. Consequently, as the level of synchronization required increases, the feasibility of process virtualization decreases. Overby and Konsynski (2010) showed that synchronization demand had a negative effect on process virtualizable demand in online auction research. Balci and Rosenkranz (2014) identified the synchronization requirements as the biggest impediment to process virtuality in Germany’s online banking study. Agrawal et al. (2020) verified that converting the working process to a virtual environment would be difficult as the synchronization requests of organizational members increased. In this study, we derived the following research hypothesis by referring to previous research results and the characteristics of telecommuting: synchronism requirements hurt the virtuality of the process.

H3: Synchronism requirements negatively impact business process virtualizability.

### Information system’s negative effect and business process virtualizability

Information overload refers to a situation where people receive more information than they can process and utilize the information, leading to anxiety or a negative sense of failure (Granero-Gallegos et al., 2021). Information overload affects information-intensive industries across society, including government, education, family life, online consumption, and citizenship (Bawden and Robinson, 2009; Huang and Wang, 2022). In the digital information age, individuals and organizations are overwhelmed by too much information. Because there is too much information to read and process, which reduces productivity and performance, and is not conducive to business innovation (Bawden and Robinson, 2009; Jackson and Farzaneh, 2012). Based on the media richness theory, Hwang et al. (2020) demonstrated that the information overload caused by instant messaging affects civil servants’ work engagement. Yu et al. (2018) explained how excessive use of social media at work affects individual work performance through an extended stressor–strain–outcome research model. Its results prove that information overload is a vital stress factor affecting social media burnout, and social media burnout further significantly reduces individual job performance.

In our study, organization members worked from home through communication technology-based information systems, and business processes eliminated physical interaction, which led to a substantial increase in the information exchanged online. In this virtualized work environment, when teleworkers search for information through collaborative software to process business and make decisions, individuals can obtain deeper and broader information than necessary (Landers and Schmidt, 2016). This process too much information requires individuals to spend a lot of time and effort to extract the information that is useful to them. Thus, we speculate that information overload prevents the business process virtualizability.

H4: Information overload negatively impacts business process virtualizability.

Communication overload refers to the network’s communication demand exceeding the individual’s communication ability (Barrett et al., 2022). Too many emails, phone calls, text messages, instant messages, and social media notifications can interfere with people’s everyday life and work (Lin et al., 2021). Through collaboration software like DingTalk, team members in the virtual workplace will inevitably receive more notifications. Organization members frequently interrupt their work due to various notifications, making it difficult for them to concentrate, which can cause organization members to become tired or anxious (Granero-Gallegos et al., 2021). Not only that, but it forces organization members to multitask because the effort is spent contacting people *via* different methods while working, which can be distracting (Reinecke et al., 2017). The distractions threaten the work output of members of the organization. According to a University of California Irvine study, “it takes an average of 23 min and 15 s to get back to the task.” Even if members of an organization eliminate distractions, they will not be as productive as they were before the distractions (Mark et al., 2008). Therefore, we deduce the following hypothesis.

H5: Communication overload negatively impacts business process virtualizability.

**FIGURE 1:**
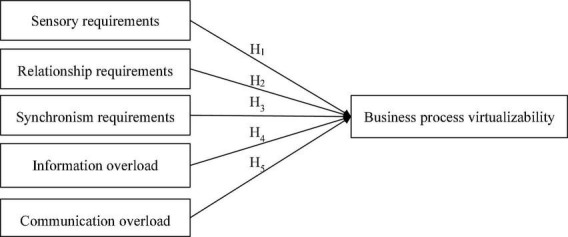
The conceptual model.

We review the PVT literature and propose a model suitable for studying business processes. To understand the relationship between several variables, we proposed and examined several hypotheses in Figure 1.

## Research methodology

### Sample and procedure

This study selected the fully mandatory teleworkers during the COVID-19 pandemic as study subjects. To prevent the spread of the epidemic, China has effectively implemented government-led regional isolation and social distancing policies. Therefore, many members of the organization began to use collaboration software (DingTalk) to work remotely, which provided an opportunity for our research. Unlike previous hybrid telecommuting, the physical interaction between teleworkers during the epidemic is wholly eliminated, which benefits this study of business process virtualization.

Due to the restrictions of the quarantine policy, starting from October 2021, 1 month period, we conducted a questionnaire survey online using a random sampling method. The questionnaire survey was managed through China’s “Wen Juan Wang” online survey platform. A total of 380 samples were collected from teleworkers. After excluding 37 invalid samples, the remaining 343 samples were used for data analysis. Those who participated in the survey were telecommuters using Alibaba’s DingTalk system. They are mainly engaged in e-commerce, dealing in food, clothing, and so on related to infants and young children. According to “The 47th China Statistical Report on Internet Development 2021” published by the China Internet Network Information Center (CNNIC), there were approximately 5 million teleworkers in China at the time of this survey. Based on the calculation of the number of respondents and population size, the margin of error in this paper is 5.29%.

Table 1 reflects that males accounted for 42.86%, and females accounted for 57.14%. People in their 20 s accounted for the most, accounting for 63.55%, followed by people in their 30 s, accounting for 24.78%. Among the number of employees working in the company, 58.35% had less than 50 employees, 25.19% had 50–99 employees, and 16.47% had more than 100 employees.

**TABLE 1:** Descriptive statistics.

Variable	Type	No. of people	Proportion
Gender	Male	147	42.86%
	Female	196	57.14%
Age	20–30	218	63.55%
	30–40	85	24.78%
	40–50	29	8.45%
	>50	11	3.22%
Education	Specialist university	42	12.24%
	Local university	225	65.59%
	Master’s degree	53	15.45%
	Doctoral degree	23	7.71%
Company size	<50 people	201	58.34%
	50–99 people	86	25.19%
	>100 people	56	16.47%

### Measures

The study used a five-point Likert scale (1 = very unimportant to 5 = very important) to record responses. First, we conducted a test before the formal investigation to verify the questionnaire’s feasibility. After analyzing and discussing the test results with relevant experts, we modified the questions, wording, and ambiguity of the questionnaire and finally formed a 21-item questionnaire. The questionnaire was distributed as a formal questionnaire (Table 2). Sensory, relationship, synchronism requirements, and business process virtualizability were measured using 14 items from Overby (2008) and Alarabiat et al. (2023). Information overload and communication overload were measured using seven items from Granero-Gallegos et al. (2021).

**TABLE 2:** Reliability tests for constructs and items.

Variables	Items	Outer loadings	rho_A	AVE	Adj *R*^2^	*Q* ^2^
Sensory requirements	In the traditional face-to-face working process in the company… V1-1: I like to see, walk through, and setting in the company/office	0.787	0.827	0.652	–	–
	V1-2: I like to see and hear my colleagues	0.813				
	V1-3: I appreciate/value the sensation of the objects (Office environment, desk chairs, etc.)	0.795				
	V1-4: In general, I like and admire the feeling of being at the company/office	0.835				
Relationship requirements	V2-1: I like to talk and establish a personal friendship with my colleagues or my boss	0.870	0.876	0.673	–	–
	V2-2: I like/enjoy social aspects with my colleagues in leisure facilities outside the company/office such as restaurants or coffee shops	0.823				
	V2-3: Overall, I enjoy the social aspects of being at the company/office	0.765				
Synchronism requirements	V3-1: To work better, I think it is necessary to have immediate access to my colleagues or supervisors.	0.685	0.843	0.621	–	–
	V3-2: To work better, I feel the need to get immediate responses from colleagues or supervisors	0.892				
	V3-3: To work better, I need to know the progress of my colleagues’ business immediately	0.773				
Information overload	When I use Ding Talk to work remotely… V4-1: I think the Ding Talk gives me too much information	0.862	0.854	0.768	–	–
	V4-2: I feel receiving a lot of unnecessary information	0.861				
	V4-3: The amount of information the Ding Talk pushes to me and others is inconsistent	0.905				
Communication overload	When I use Ding Talk to work remotely… V5-1: I often receive more information than I can handle	0.933	0.932	0.879	–	–
	V5-2: I often send more messages than I expect	0.925				
	V5-3: Too many messages and notifications have been interrupting my daily work now	0.954				
Business process virtualizability	V6-1: I can handle the job at hand quickly	0.865	0.835	0.627	0.163	0.142
	V6-2: My productivity has increased	0.847				
	V6-3: I can handle some urgent work well	0.711				
	V6-4: I found a better way of doing business	0.733				

### Data analysis

To ensure the robustness and validity of the theoretical hypotheses proposed in this study, a structural equation modeling (SEM) approach was employed to analyze the data. This method allows for the examination of complex relationships among multiple variables, which is particularly useful in light of the multiple factors that may influence the model under examination.

To facilitate this analysis, the SmartPLS 4 program was utilized. This software is widely used in various fields and employs partial least squares (PLS) SEM to analyze structural equations (Ali Qalati et al., 2020). The program is known for its comprehensiveness, with an intuitive graphical interface that allows for the observation of interrelationships among multiple variables (Hair et al., 2011, 2019).

Furthermore, the measurement model and structural model were evaluated using an improved metric proposed by Hair et al. (2011). This new metric not only assesses internal consistency and discriminative validity but also effectively validates the predictive ability of the structural model (Sarstedt et al., 2022).

## Results

### Measurement model

The present study employs SEM and PLS techniques as outlined in Sarstedt et al. (2022) to examine the measurement models. The results, presented in Table 2, demonstrate that the constructs possess high levels of reliability, as evidenced by the Cronbach’s Alpha and rho_A values, which are greater than or equal to 0.7. Additionally, the Mean-Variance Extracted values (AVE) are greater than or equal to 0.5, indicating strong convergent validity (Sarstedt et al., 2022). Furthermore, in accordance with the guidelines provided by Franke and Sarstedt (2019), this study evaluates the discriminant validity of the constructs by utilizing the heterotrait-monotrait ratio (HTMT) method, as presented in Table 3. The results indicate that all HTMT critical values are less than 0.9, indicating that the constructs possess strong discriminant validity.

**TABLE 3:** Discriminant validity.

Relationship between construct	Heterotrait-monotrait ratio (HTMT)
Communication overload < - > Business process virtualizability	0.237
Information overload < - > Business process virtualizability	0.178
Information overload < - > Communication overload	0.072
Relationship requirements < - > Business process virtualizability	0.293
Relationship requirements < - > Communication overload	0.104
Relationship requirements < - > Information overload	0.430
Sensory requirements < - > Business process virtualizability	0.296
Sensory requirements < - > Communication overload	0.121
Sensory requirements < - > Information overload	0.144
Sensory requirements < - > Relationship requirements	0.593
Synchronism requirements < - > Business process virtualizability	0.266
Synchronism requirements < - > Communication overload	0.118
Synchronism requirements < - > Information overload	0.174
Synchronism requirements < - > Relationship requirements	0.691
Synchronism requirements < - > Sensory requirements	0.422

### Structural model

**FIGURE 2:**
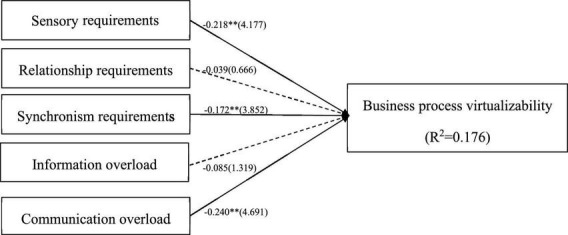
Result of path analysis. *p-value < 0.05; **p-value < 0.01.

The value of *R*^2^ represents the explanatory power of the PLS structural model. The explained variance is shown in Figure 2. All independent variables of this study can explain 17.6% of business process virtuality. The value of *R*^2^ ranges from 0 to 1, and the larger the value, the stronger the explanatory ability of the structural model. According to Raithel et al. (2012)’s claim, the acceptable *R*^2^ value is above 0.1. The coefficients for the structural relationships among all variables were obtained by computing a series of regression equations. In addition, we increased the value of *Q*^2^ (Table 2) in this study to evaluate the prediction accuracy of the PLS path model. A higher *Q*^2^ value means higher prediction accuracy (Pangarso et al., 2022). Before assessing structural relationships, collinearity must be checked to ensure that it does not affect the results obtained from the regression equation. The collinearity of all formed indicator sets can be assessed by calculating the variance inflation factor (VIF).

Hair et al. (2019) as shown in Table 4, the VIF values of this survey are all less than 3, which proves that there are no collinearity issues. This study calculated the Model fit of the model using SRMR (Hair et al., 2021). Table 5 shows that the model structure is a fit model with the SRMR, d_ULS and d_G values already at the 95% confidence interval. This study uses SmartPLS 4 to calculate the path coefficients and test the hypotheses. Table 6 shows the significant results using normalized path coefficients (β-values) and *t*-values of path coefficients. Among them, H1 (β = −0.218, *t* = 4.177), H3 (β = −0.172, *t* = 3.852), and H5 (β = −0.240, *t* = 4.691) passed the significance test. The results can be interpreted to conclude that sensory requirements, synchronism requirements, and communication overload significantly negatively impact process virtualizability. However, the path coefficient of H2 was −0.039 (*t* = 0.666), and H4 was −0.085 (*t* = 1.319), which proves that relationship requirements and information overload are not the influencing factors that hinder business process virtualization.

**TABLE 4:** Collinearity statistics variance inflation factor (VIF).

	Business process virtualizability
Business process virtualizability	–
Communication overload	1.052
Information overload	1.135
Relationship requirements	1.777
Sensory requirements	1.321
Synchronism requirements	1.334

**TABLE 5:** Model fit.

	Saturated model	Estimated model
SRMR	0.084	0.084
d_ULS	1.473	1.473
d_G	0.629	0.629
NFI	0.676	0.676

**TABLE 6:** Path coefficients and hypothesis testing.

Hypothesis	Relationship	β -value	*t*-value	Decision
H1	Sensory requirements → Process virtualizability	−0.221	4.251**	Supported
H2	Relationship requirements → Process virtualizability	−0.027	0.683	Not supported
H3	Synchronism requirements → Process virtualizability	−0.205	4.338**	Supported
H4	Information overload → Process virtualizability	−0.090	1.558	Not supported
H5	Communication overload → Process virtualizability	−0.229	4.834**	Supported

Path coefficient significance level *p-value < 0.05; **p-value < 0.01.

## Discussion

This study verifies five variables that may hinder business process virtualization. The results show that sensory requirements, synchronism requirements, and communication overload negatively impact process virtualizability. This finding is consistent with the results of existing literature research. For example, the higher the sensory and synchronism requirements, the lower the virtualizability of the online banking transaction process and the second-hand transaction process (Overby and Konsynski, 2010; Balci and Rosenkranz, 2014). Additionally, numerous studies have documented communication overload as a contributing factor to the inefficiency of telecommuting (Granero-Gallegos et al., 2021; Barrett et al., 2022). Namely, communication overload hinders the virtualization of business processes. In this study, Business processes migrate from a physical environment to a virtual environment based on information systems, and the physical interactions are wholly eliminated. So, the communication between members of an organization is entirely dependent on information systems. As a result, members of the organization can only obtain information through a single sense, which is not conducive to communication, and cannot provide people with the convenience and comfort obtained through multiple senses (Alarabiat et al., 2023). In addition, the communication between organization members needs to improve immediacy, and teleworkers cannot immediately communicate with team members on urgent business, leading to a decline in business results. At the same time, excessive notifications can also cause business disruption for other employees, low concentration, and ultimately negatively affect the business process. Businesses and employees can balance synchronism requirements and communication overload to maximize process virtualizability and business outcomes.

However, we find that relational requirements and information overload are no longer barriers to business process virtualizability. This result differs from previous research on PVT (Barth and Veit, 2011; Swar et al., 2017; Granero-Gallegos et al., 2021; Alarabiat et al., 2023). Existing literature proves that the higher the relationship requirements and information overload of the participants in a process, the lower the virtualizability of the process. For example, citizens with higher relational requirements are more resistant to e-government service systems (Barth and Veit, 2011). And the higher the relationship requirements of the students, the lower the distance learning outcomes (Alarabiat et al., 2023). Likewise, information overload is responsible for poor process virtualization (Swar et al., 2017; Granero-Gallegos et al., 2021). The results of this study did not reveal a significant relationship between relationship requirements and process virtualizability. One possible explanation for this is that the measures used to assess relationship requirements and process virtualizability may not have been appropriate for the population or setting studied. Specifically, remote workers in the COVID-19 environment may have different ways of building and maintaining relationships, as they are more likely to use online communication tools to protect themselves and their families. Therefore, future research could investigate the relationship between relationship requirements and process virtualizability by using more appropriate measures and taking into consideration the unique context of remote work during the COVID-19 pandemic. In addition to the possibility previously discussed, another potential explanation for the lack of significant relationship between relationship requirements and process virtualizability is that the relationships established through collaboration software may be sufficient to meet the relationship requirements required in business processes. Utilization of work-oriented collaboration software can bring colleagues who are already familiar with each other closer, while social-oriented use can facilitate the formation of relationships among individuals or groups across departments who may not have previously known one another (Baker and Dutton, 2017). Furthermore, the formation of a tight-knit network of relationships through collaboration software can facilitate the flow of resources and knowledge within an organization (Baker and Dutton, 2017). From the perspective of social capital, collaboration software can provide organizations and employees with ample opportunities to build social connections in the workplace, which can be beneficial for employees to accumulate social capital (Fu et al., 2019).

In addition, the results of this study demonstrate that information overload does not negatively affect business process virtualization. This result is not consistent with our previous hypothesis. From the perspective of organizational behavior, collaboration software has personal social-oriented functions and work-oriented enterprise social functions (Van Zoonen et al., 2017). Through personal social-oriented functions, people receive much information of poor quality (Granero-Gallegos et al., 2021), and it takes time for the information receiver to determine the authenticity of the information. Negative emotions will arise when people spend much time obtaining adequate information (Naveed and Anwar, 2020). The main reason people experience anxiety from information overload is the inability to filter out reliable and valuable information from a large amount of information (Swar et al., 2017). However, unlike personal social-oriented functions, work-oriented enterprise social functions are limited to employees of the organization because the quality of information exchanged through corporate social media is relatively higher due to organizational norms and policies (Leonardi et al., 2013). There is a positive impact on member productivity (Leftheriotis and Giannakos, 2014). Although there is too much information exchanged through collaboration software, members of the organization can spend less energy and time sifting through this information, so the information overload will not affect the organization’s work efficiency.

### Theoretical contribution

This study applies the PVT to telecommuting and contributes to expanding the application. Moreover, we proved that some variables in the prior research are no longer applicable to business processes, providing new evidence for business-related researchers to study business processes. The core contribution of this study is that we reveal the harmful effects of information systems on business processes that have been neglected in previous studies based on PVT and effectively enhance the explainability of PVT on business processes.

Additionally, this research provides a timely contribution. Organization members have been working remotely for extended periods due to COVID-19. Unlike previous occasional remote or hybrid telecommuting, mandatory isolation policies keep members of the organization in a virtual space with no physical interaction, providing a better environment for virtualizing the research process. Excluding the influence of other factors provides a better look at what hinders the migration of business processes from physical to virtual space. The findings could help future researchers study how organizations implement telecommuting.

### Practical implications

This study reveals the factors that hinder the business process virtualization and provides a basis for enterprises to implement teleworking. To promote enterprises’ digital transformation and increase business process virtualization. Governments and businesses can develop guidance and policies on using information systems and clarify when information is exchanged between team members. Find a balance that ensures efficient collaboration without letting too much communication disrupt their work. Managers can control information access and distribution by integrating knowledge exchange processes and the social activities of teleworkers in virtual environments. On an individual level, teleworkers can create an office-like environment to satisfy their senses. Schedule management through collaboration software, prioritize communication with teams, and reduce distractions caused by notification reminders.

### Limitations and future research

Although our research reveals essential discoveries, there are also limitations. Our research only collected data on the use of DingTalk for telecommuting. DingTalk is the most used collaboration software among Chinese enterprises, so a single type of collaboration software limits the applicability of our results to different information systems. Future research can adopt a cross-country, cross-cultural approach to study business process virtualization based on different information systems. Improve the accuracy of research results by expanding the scope of the survey and increasing the sample size.

## Conclusion

From the above discussion, the conclusion can be reached that the factors hindering the business process virtualizability have changed. Scholars have re-examined process virtualizability in recent years because of the COVID-19 pandemic. Because of the differences in process characteristics and the perfection of information systems, there are differences in the factors affecting process virtualization in different virtual processes. Our research aims to examine what factors hinder business process virtualization, not to negate previous research. Especially in the specific context of COVID-19, teleworkers can only use information systems to conduct business. Fully virtualized business processes are more conducive to studying the psychological requirements of teleworkers and their impact. The results of this study not only bridge the previous theoretical gap but also provide a new perspective for studying business processes. In the future, in the so-called new “normal era,” our research helps companies work more efficiently in the virtual space based on information systems to improve employee productivity and the competitiveness of companies in the rapidly changing business environment.

## Data availability statement

The raw data supporting the conclusions of this article will be made available by the authors, without undue reservation.

## Ethics statement

This study does not require ethical review and approval following local legal and institutional requirements. We provided written informed consent to those who participated in the investigation. The participants provided their written informed consent to participate in this study.

## Author contributions

YF contributed to all the phases of the study from conception and design of the study, statistical analysis, results interpretation, contributed to theoretical literature review, data collection, and writing the first draft. JP contributed to supervision and the revision of the work. MF contributed to conception of the study and data collection. All authors discussed the results and contributed to the final manuscript.
